# The Role of Metabolic Reprogramming in the Tumor Immune Microenvironment: Mechanisms and Opportunities for Immunotherapy in Hepatocellular Carcinoma

**DOI:** 10.3390/ijms25115584

**Published:** 2024-05-21

**Authors:** Nan Hu, Haiyang Li, Changcheng Tao, Ting Xiao, Weiqi Rong

**Affiliations:** 1 Department of Hepatobiliary Surgery, National Cancer Center/National Clinical Research Center for Cancer/Cancer Hospital, Chinese Academy of Medical Sciences and Peking Union Medical College, Beijing 100021, China; dr_hunan01@126.com (N.H.); li_haiyang0708@126.com (H.L.); 15601170661@163.com (C.T.); 2 State Key Laboratory of Molecular Oncology, Department of Etiology and Carcinogenesis, National Cancer Center/National Clinical Research Center for Cancer/Cancer Hospital, Chinese Academy of Medical Sciences and Peking Union Medical College, Beijing 100021, China

**Keywords:** hepatocellular carcinoma, metabolic reprogramming, tumor microenvironment, immunotherapy, metabolic intervention

## Abstract

As one of the emerging hallmarks of tumorigenesis and tumor progression, metabolic remodeling is common in the tumor microenvironment. Hepatocellular carcinoma (HCC) is the third leading cause of global tumor-related mortality, causing a series of metabolic alterations in response to nutrient availability and consumption to fulfill the demands of biosynthesis and carcinogenesis. Despite the efficacy of immunotherapy in treating HCC, the response rate remains unsatisfactory. Recently, research has focused on metabolic reprogramming and its effects on the immune state of the tumor microenvironment, and immune response rate. In this review, we delineate the metabolic reprogramming observed in HCC and its influence on the tumor immune microenvironment. We discuss strategies aimed at enhancing response rates and overcoming immune resistance through metabolic interventions, focusing on targeting glucose, lipid, or amino acid metabolism, as well as systemic regulation.

## 1. Introduction

As the largest gland in the human body, the liver is the centre of metabolic regulation. It mediates the metabolism of macro- and micronutrients, including carbohydrates, lipids, proteins, hormones, bile, and xenobiotics, which are responsible for maintaining metabolic homeostasis and regulating disease progression [1,2]. However, influenced by risk factors such as chronic hepatitis B or C virus infection, excessive alcohol consumption, or nonalcoholic fatty liver disease (NAFLD), the normal liver structure can be disrupted, leading to chronic liver inflammation, fibrosis, and cirrhosis, in which aberrant metabolic remodeling and liver carcinogenesis can occur [1,3]. Additionally, the repeated regenerative repair of hepatocytes facilitates the accumulation of gene mutations, including ras, p21, or p53, which are precursors to hepatocellular carcinoma (HCC) [4,5]. As the most common type of primary liver cancer, HCC ranks third among the dominant causes of tumor-related mortality worldwide [6].

The majority of patients (approximately 65–70%) are diagnosed with HCC at intermediate or advanced stages, thus missing the opportunity to receive radical resection or liver transplantation [7]. Due to the critical role of the immune system, immunotherapy has proven effective in various clinical trials for restoring innate and adaptive immunity and reversing the immunosuppressive tumor microenvironment (TME). Immune checkpoint inhibition (ICI) has also been emphasized [5,8]. Despite remarkable breakthroughs, objective response rates (ORRs) ranging from 15–20% have been reported from multiple single-agent studies of anti-cytotoxic T lymphocyte-associated protein 4 (CTLA4) or anti-programmed cell death 1/ligand 1 (PD-1/PD-L1) inhibitors [9]. Due to single-agent ICIs’ poor ability to increase overall survival (OS), combined strategies, such as the combination of atezolizumab and bevacizumab, have been proposed and evaluated in clinical trials [9]. Combining immunotherapy with other strategies may constitute a useful method for treating HCC, as complex mechanisms and efficacy-determining factors underlie ICI resistance, including metabolic reprogramming of the HCC TME has garnered increasing attention [7,10,11].

Metabolic reprogramming of the HCC TME has been widely investigated, mostly in preclinical trials; it is characterized by dysregulated glycolysis, lipogenesis, glutamine addiction, and regional metabolic zonation in specific anatomical locations. The TME in HCC is a complex and immunosuppressive milieu consisting of cellular components (immune cells and stromal cells), extracellular matrices (ECMs), and substances from exosomes and secretomes, such as soluble cytokines [8,12]. The TME is commonly hypoxic and acidic, with abnormal angiogenesis and an irregular ECM; it also promotes the growth and invasion of tumor cells. Cellular composites, including regulatory T-cells (Tregs), macrophages, myeloid-derived suppressor cells (MDSCs), cancer-associated fibroblasts (CAFs), and immature dendritic cells (DCs), participate in promoting immune tolerance [8]. Upon metabolic remodeling of the TME, cells react distinctively to nutrient competition. For instance, nutrient deprivation and accumulation of harmful metabolic intermediates can impair the anti-tumor functions of immune cells, which is closely related to the remodeling of tumor-immune landscapes [13].

In this review, we characterize the metabolic state of HCC and elucidate its influence on the immune state of the TME and response to ICIs. Furthermore, trials aimed at enhancing immunologic function or reversing ICI resistance via metabolic intervention are also presented.

## 2. Impact of Dysregulated Glucose Metabolism in the TME

HCC cells reprogram metabolic patterns by adjusting the levels of relevant enzymes and transporting proteins to support glucose uptake, glycolysis, and the discharge of metabolic products, leading to intracellular lactate accumulation and nutrient shortages [14]. Carbohydrate metabolism reprogramming begins at HCC initiation, during which glycolysis, the tricarboxylic acid cycle (TCA cycle), gluconeogenesis, and the pentose phosphate pathway (PPP) are involved, profoundly influencing the immune landscape.

Metabolic alterations in the glycolytic pathway are significant and versatile, involving alterations in various metabolic enzymes and bioactive intermediates. Tumor cells accelerate glycolysis regardless of oxygen availability, which is also called the Warburg effect [2]. With a median oxygen level of 0.8% due to rapid oxygen consumption and abnormal angiogenesis, hypoxia activates the hypoxia-inducible factor (HIF) and upregulates PD-L1 expression. In combination with the lactate-TGF-β signaling pathway, PD-L1 facilitates escape from immune surveillance [15]. Pyruvate kinase isozyme type M2 (PKM2) upregulates HIF-1α, Gli1, and Bcl-xl expression, enhances cellular proliferation, and PKM2 inhibition restrains glucose uptake. HIF-1α impedes immune surveillance and natural killer (NK) cell infiltration by interfering with MHC class I polypeptide-related sequence A (MICA) and inhibiting the expression of natural cytotoxic receptors and the activator NKG2D, thereby interrupting tumor antigen recognition [15,16]. Perforin, interferon-γ (IFN-γ), and granzyme B secretion is also negatively associated with NKp46 downregulation [17,18].

**Figure 1:**
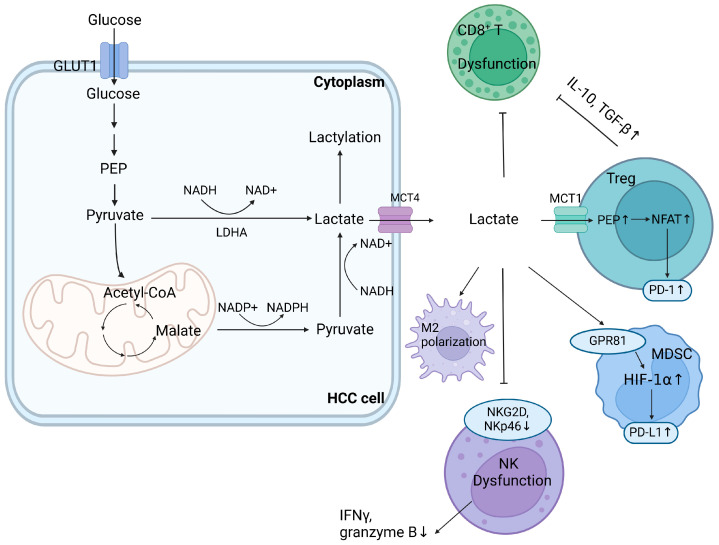
Impact of lactate accumulation from dysregulated glucose metabolism on the TME. Accumulated lactate from glucose metabolism in TME promotes immunosuppression through various mechanisms. It enhances Treg activity by facilitating NFAT1 translocation into the nucleus and increasing lactate absorption, suppressing CD8+ T-cell function by upregulating IL-10 and TGF-β. Lactate boosts MDSC generation via the GPR81/HIF-1α/PD-L1 axis, leading to PD-L1 upregulation. This axis also inhibits NK cell infiltration and cytotoxicity by downregulating natural cytotoxic receptors NKG2D and NKp46 and cytotoxic molecule secretion. In addition, lactate induces M2 polarization. GLUT1, glucose transporter type 1; HCC, hepatocellular carcinoma; HIF-1α, hypoxia-inducible factor 1α; LDHA, lactate dehydrogenase A; MDSCs, myeloid-derived suppressor cells; NAD/NADH, nicotinamide adenine dinucleotide; NADP/NADPH, nicotinamide adenine dinucleotide phosphate; NK, nature killer; PEP, phosphoenolpyruvate; TGF-β, transforming growth factor-β; Treg, regulatory T-cell.

Lactate accumulation from increased glycolysis leads to TME acidification and disrupts lactate homeostasis under the synergetic influence of oncogenes such as HIF-1, c-myc, and PI3K/AKT [19]. Lactate increases IFN-γ secretion, leading to T-cell exhaustion via the PD-1/PD-L1 pathway. However, abundant lactate impedes the anti-tumor effects of cytotoxic T lymphocytes (CTLs) by blocking the import transporter MCT-1 while enhancing Tregs by facilitating NFAT1 translocation into the nucleus and augmenting lactate absorption via the upregulation of MCT-1 and lactate dehydrogenase A (LDHA) expression [20]. Thus, enriched Tregs inhibit the release of cytotoxic granzymes and perforin from CD8+ T-cells. They use lactate to support the TCA cycle, mediated by increasing expressions of IL-10 and TGF-β, maintaining proliferation even under hypoglycemic and acidic conditions. In addition, lactate accumulation enhances IL-6- and GM-CSF-regulated MDSC generation possibly via the Gi-protein-coupled receptor 81 (GPR81)/mTOR/HIF-1α/STAT3 axis [21,22]. By enhancing MCT-4 expression and the glycolytic rate, lactate promotes M2 polarization by inducing arginase-1 (Arg1), HIF-1α, IL-6, and transcriptional repressors such as ICER, which negatively influence anti-tumor responses [23]. When stimulated by glycolysis and fibronectin 1 (FN-1), HLA-DR- and CD86-high macrophages exhibit a glycolytic phenotype, impeding the secretion of the anti-tumor agent IL-12 p70 through the PKM2/HIF-1α axis [24,25]. Furthermore, lactate guides the post-translational modification of histones and lysine lactylation and regulates critical metabolic enzymes. K28 lactylation suppresses adenylate kinase 2 (AK2), promoting HCC proliferation and metastasis [26]. Figure 1 provides a concise overview of the impact of lactate accumulation from glucose metabolism in the TME on immune evasion.

Apart from lactate, other intermediates from carbohydrate metabolism have also been verified to be closely related to the immune landscape of the TME. For instance, a metabolic intermediate of the TCA-cycle, itaconate, which is synthesized from the decarboxylation of TCA-cycle-derived cis-aconitate, has an overall anti-inflammatory effect and mediates metabolic interactions between cellular components in the HCC TME [27]. Itaconate has been shown to induce CD8+T exhaustion, attenuate CD8+T-cell proliferation and function, and interfere with myeloid immune cells’ normal functions [27]. The synthesis of itaconate is regulated by the mitochondrial enzyme immune-responsive gene 1 (IRG1), also referred to as aconitate decarboxylase 1 (ACOD1). Increased expression of IRG1 and itaconate derived from peritoneal tissue-resident macrophages promotes peritoneal tumor progression by increasing ROS generation in macrophages and activating the MAPK signaling pathway [27,28]. In addition, itaconate has been shown to mediate glycolysis, inhibit succinate dehydrogenase (SDH), and activate the transcription factors Nrf2 and ATF3, which are involved in the accumulation of succinate-mediated H3K4me3 and the transcriptional modulation of exhaustion markers PD-1 and TIM-3. These findings suggest itaconate’s potential as an immunometabolite target for immune modulation of the TME [28,29].

To sustain ribonucleotide synthesis and redox balance, PPP is activated to replenish nicotinamide adenine nucleotide phosphate (NADPH) production to escape oxidative stress [30], with upregulated expression of critical enzymes such as glucose-6-phosphate dehydrogenase (G6PD) or transketolase [31]. However, gluconeogenesis-related enzymes such as fructose-1,6-bisphosphatase 1 (FBP1) and phosphoenolpyruvate carboxykinase 1 (PCK1) are downregulated in HCC cells. Forced PCK1 expression in HCC cell lines leads to oxidative damage, apoptosis, and inhibition of liver carcinogenesis [32,33]. Besides, FBP1 downregulation in hepatocytes is associated with reduced PKLR expression in the extracellular vesicles (EVs) they release. Notably, NK cells are prominent recipients of EVs derived from hepatocytes. Due to PKLR’s role in promoting glycolysis through the conversion of phosphoenolpyruvate to pyruvate, depletion of FBP1 and the subsequent absence of PKLR in EVs impair nutrient availability and compromise the tumor surveillance function of NK cells [34].

In general, dysregulated carbohydrate metabolism, characterized by a substantial increase in glycolysis, a decrease in gluconeogenesis, and altered levels of intermediates such as lactate and itaconate, has versatile and mostly unfavorable effects on the immunologic landscape of HCC TMEs. It dampens the cytotoxic functions of NK cells, CTLs, and macrophages while enhancing the immunosuppressive effects of Tregs, MDSCs, and M2 polarization. In addition, diverse alterations in glucose metabolic pathways during carcinogenesis present potential targets for metabolic intervention such as critical rate-limiting enzymes or intermediates.

## 3. Impact of Dysregulated Lipid Metabolism in the TME

Due to an insufficient nutrient supply, the gradual transition of liver tissue from precancerous to HCC tissue is accompanied by a switch from glucose-dependent to lipid-dependent metabolism. In addition to serving as alternative energy sources for cells withstanding cellular proliferation and growth, satisfying the need for energy, and supplying materials for membrane formation, lipids can also mediate post-translational modifications and function as secondary messengers to regulate oncogenic transcription factors, such as HIF-1α, NF-κB, STAT3, and AP-1, or signaling pathways such as the TGF-β and Wnt [1].

Altered lipogenesis and lipid accumulation are common in HCC TMEs and differentially influence the immune functions of cellular components. Lipid lipase (LPL), which breaks down triacylglycerols into fatty acids (FAs), is upregulated in HCC tumor cells to increase exogenous lipid transportation and absorption. It also increases CD36 expression, a FA transporter [35,36]. In addition, increased lipogenesis in HCC is characterized by increased expression of metabolic enzymes, including ATP citrate lyase (ACLY), fatty acid synthase (FASN), and acetyl-CoA carboxylase (ACC) [35]. Upregulated de novo FA synthesis or oxidized FAs from CTNNB1-mutated HCC cells are vital energy sources for metabolic reprogramming [37].

Nevertheless, pathological accumulation of FAs via fatty acid synthesis (FAS) exacerbates liver inflammation. The accumulation of palmitic acid (PA), a long-chain saturated fatty acid, in Hep3B cells upregulates TGF-β1 expression and colony-stimulating factor 1 (CSF1), enhancing the polarization of M2 tumor-associated macrophages (TAMs) and the immunosuppressive phenotype of CAFs [38]. However, the effects of FAs on T-cells are concentration-dependent, with low FA concentrations affecting proliferation, whereas high FA concentrations can result in dysfunction and apoptosis via lipotoxicity [37]. In this way, FA catabolism can utilize FAs and heighten the immune response. However, in DCs, FA synthesis is attenuated through the inhibition of mTORC1 or sterol regulatory element-binding protein 1 (SREBP-1), the master regulator of ACC, SCD, and FASN [38,39]. Downregulated FA synthesis is partially mediated by α-fetoprotein (AFP), which originates from HCC cells and is a typical serum biomarker for HCC [40]. AFP-mediated decreases in FA synthesis are detrimental to DC differentiation and growth, leading to disrupted antigen processing, presentation, and immune response initiation [39]. Therefore, the differential effects of dysregulated FAS and FA accumulation on specific cellular components suggest the complexity of metabolic and immune regulation in HCC TMEs. In addition, they highlight the need for accurate targeting to modulate immune functions.

**Figure 2:**
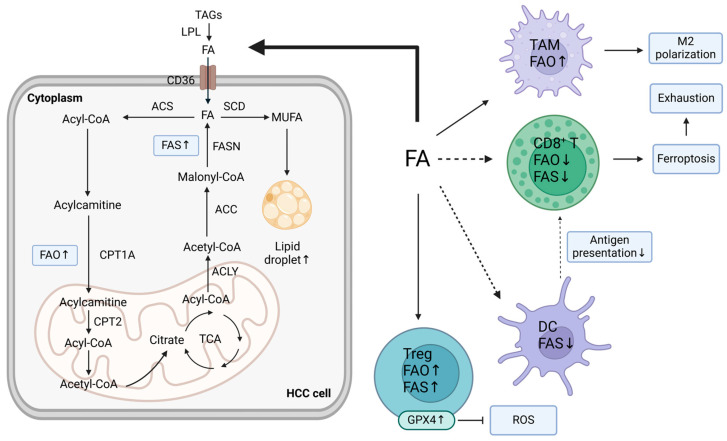
The impact of fatty acid uptake competition and lipid metabolism reprogramming on the TME. Lipids serve as alternative energy sources for tumors and immune cells, impacting the immunosuppressive TME. In HCC, increased lipogenesis, marked by elevated metabolic enzymes, provides vital energy sources for tumor survival and proliferation. Accumulated FAs promote M2 TAM polarization. Reduced FA synthesis impairs DC function, disrupting antigen processing and immune response initiation. Tregs with high GPX4 levels exhibit enhanced nutrient utilization and higher FAS and FAO rates, while CD8+ T-cells succumb to oxidative stress, leading to T-cell exhaustion and ferroptosis. ACC, acetyl-CoA carboxylase; ACLY, ATP citrate lyase; CPT1A, carnitine palmitoyl-transferase 1A; CPT2, carnitine palmitoyl-transferase 2; DC, dendritic cell; FA, fatty acid; FAO, fatty acid oxidation; FAS, fatty acid synthesis; FASN, fatty acid synthase; GPX4, glutathione peroxidase 4; HCC cell, hepatocellular carcinoma cell; IFN-γ, interferon-γ; MUFA, monounsaturated Fatty Acid; TAGs, triacylglycerols; TCA, tricarboxylic acid; Treg, regulatory T-cell. The dashed line indicates reduced uptake or diminished promotive effect.

Moreover, dysregulated levels of fatty acid oxidation (FAO) across cells in the TME play an integral role in metabolic remodeling, accompanied by the modulated expression of carnitine palmitoyl-transferase (CPT), which transports acyl-CoA into mitochondria [1,41]. In β-catenin-activated HCC, FAO is an indispensable energy resource for tumor cells with increasing peroxisome proliferator-activated receptors (PPARs) and CPT2 expression. However, lipid peroxidation, which is induced by inositol-requiring protein 1α (IRE-1α)-mediated endoplasmic reticulum (ER) stress, exacerbates immune inefficiency in DCs and M2 polarization [42]. Tregs, which possess high levels of glutathione peroxidase 4 (GPX4), are more competent at utilizing nutrients and have higher FAS and FAO rates. By contrast, tumor-infiltrating lymphocytes (TILs) fail to escape oxidative stress, resulting in T-cell disability and ferroptosis [42,43]. As a vital factor in inflammation and necroptosis, receptor-interacting protein kinase 3 (RIPK3) expression is downregulated in TAMs and promotes M2 polarization. A lack of RIPK3 leads to decreased ROS and inhibits caspase1-mediated PPAR cleavage, which activates PPAR and significantly facilitates FA metabolism [44]. In this way, FAO and M2 polarization of TAMs are promoted via the RIPK3-ROS-Caspase1-PPAR pathway. By analyzing single-cell RNA sequencing (scRNA-seq) data from human and murine HCC tumors, a new type of CAF, CD36+ CAFs, was identified. These cells originate from hepatic stellate cells (HSCs) and promote lipid peroxidation, p38 kinase stimulation, and CD33+ MDSC recruitment [45]. Since most HCC cases originate from chronic liver inflammation and cirrhosis, enriched activated fibroblasts become CAFs, which secrete growth factors, collagens, matrix metalloproteinases (MMPs), and exosomes to regulate ECM integrity upon entry into tumors [46,47]. When inhibiting serum lysophosphatidic acid (LPA) through α-bromomethylene phosphonate (BrP) or gene silencing, transdifferentiation from paired peritumoral tissue fibroblasts (PTFs) to the CAF-like myofibroblastic phenotype is blocked, suggesting LPA’s critical role in mediating the transdifferentiation process [47]. Figure 2 depicts the impact of fatty acid uptake competition and lipid metabolism reprogramming on TMEs.

Therefore, in light of the versatile roles that lipids play in immune conditions and diverse alterations in lipid metabolism during liver carcinogenesis, research on strategies targeting dysregulated lipogenesis, lipid accumulation, or oxidation is promising and meaningful for regulating the immune landscape of HCC TMEs.

## 4. Impact of Dysregulated Amino Acid Metabolism in the TME

**Figure 3:**
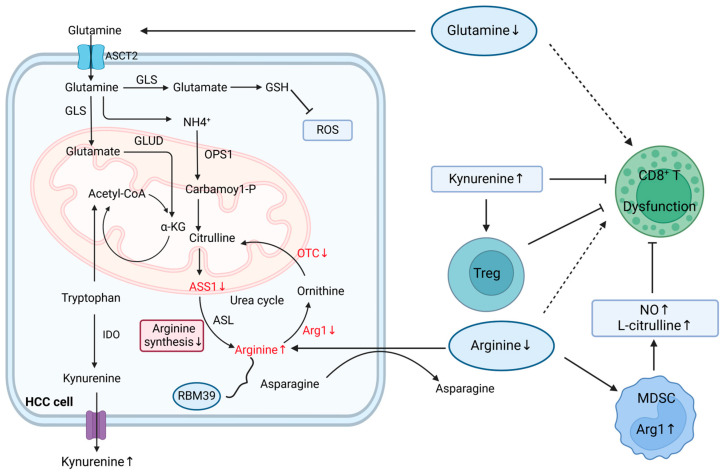
The impact of abnormal amino acid metabolism on the TME. HCC cells uptake glutamine from TME to fuel nucleotide and protein synthesis, as well as the production of antioxidant GSH. IDO catalyzes tryptophan to kynurenine transformation, depleting tryptophan and accumulating kynurenine and inducing Treg generation. Lack of ASS1 or OTC in HCC cell lines suppresses arginine synthesis. However, increased arginine import and reduced Arg1 expression elevate arginine levels in HCC cells, promoting liver carcinogenesis. Elevated arginine binds with RBM39, regulating metabolic gene expression and promoting asparagine synthesis and arginine import, creating a positive feedback loop in carcinogenesis. MDSCs secrete Arg1, degrading arginine into NO and L-citrulline, which deplete nutrients for immune cells. Overall, dysregulated amino acid metabolism drives the development of immunosuppressive TME. Arg1, arginase 1; ASCT2, alanine-serine-cysteine transporter 2; GLS, glutaminase; GSH, glutathione; HCC, hepatocellular carcinoma; IDO, indoleamine 2,3-Dioxygenase; MDSCs, myeloid-derived suppressor cells; TCA, tricarboxylic acid; Treg, regulatory T-cell; α-KG, α-ketoglutarate dehydrogenase. The dashed line indicates reduced uptake.

As another substrate nutrient source, amino acids are nitrogen and carbon donors that synthesize protein and nucleotide skeletons and maintain redox homeostasis. HCC cells take up amino acids, glucose, or ions from the extracellular domain to support the synthesis of nucleotides, proteins, and the antioxidant GSH, providing energy for the TCA cycle upon hypoglycemia [48]. The effects of abnormal amino acid metabolism on the TME are depicted in Figure 3.

HCC cells highly expressing c-myc, a transcription factor of glutaminase (GLS) and a mediator of increased glutamine uptake, tend to rely on glutamine metabolism, which is also referred to as glutamine addiction [49,50]. GLS1 enhances the proliferation and formation of HCC colonies [49], while GLS2 downregulates PI3K/AKT signaling, inhibiting cellular growth [51,52]. SLC1A5 (ASCT2) mediates glutamine transportation and enhances glutamine absorption and consumption [53]. In addition, the positive feedback loop involving Wnt, β-catenin, glutamine synthetase (GS), glutamine, and the mTOR/AKT axis further enhances GS expression and amino acid biosynthesis [49,54]. Metabolic reprogramming concurs with the remodeling of the immune subpopulation, with the immunosuppressive type CD8-Tef-APOC2 featuring the synthesis of amino acids and endogenous lipids. By contrast, the tumor-killing subpopulation CD8-Tef-GZMA depends on exogenous lipids [55,56]. Although both tumor and CD8+ T-cells depend on glutamine metabolism, disrupting glutamine via HMGB1 suppression enhances CTL infiltration and elevates the immune response due to CTL’s highly flexible metabolic dependency [51].

Other metabolites of amino acids such as indoleamine 2,3-dioxygenase (IDO) and arginine (Arg) drive the generation of immunosuppressive TMEs. IDO, which mediates the transformation from tryptophan to kynurenine and promotes tryptophan deprivation and kynurenine accumulation, induces Treg generation [12]. Arg, a highly versatile type of amino acid, not only functions as a precursor for polyamines, nitric oxide, and creatine but also differentially impacts metabolism across cellular composites in HCC TMEs [57]. The urea cycle involves the process of converting citrulline into arginosuccinate by ASS1, followed by its conversion into Arg, which is catalyzed by arginosuccinate lyase (ASL). Afterward, Arg1 degrades Arg into ornithine, which is transformed back to citrulline by OTC and then to Arg via ASS1/ASL. According to several studies, ASS1 or OTC are absent in HCC cell lines, leading to the suppression of arginine synthesis [58]. However, increasing arginine import and inhibiting Arg-to-polyamine conversion by decreasing Arg1 expression result in a high level of Arg in HCC tumor cells, which underlies liver carcinogenesis [58]. Elevated Arg remodels metabolism by binding with RNA-binding motif protein 39 (RBM39), which transcriptionally regulates metabolic gene expression, including overexpression of the gene-encoding asparagine synthetase (ASNS). Increased expression of ASNS promotes asparagine synthesis and further promotes Arg import, generating a positive feedback loop between increased Arg levels and activated carcinogenic metabolism [57]. By secreting Arg1 and nitric oxide synthase-2 (iNOS2), which further degrade L-arginine into nitric oxide (NO) and L-citrulline, respectively, MDSCs consume ingredients for protein synthesis, leading to a shortage of nutrients for immune cells, interruption of JAK-STAT signal transmission, suppression of MHC class II expression, and immune surveillance failure [59,60]. Therefore, Arg functions as a second messenger-like molecule by mediating metabolic remodeling and carcinogenesis.

In conclusion, dysregulated amino acid metabolism, as well as the expression of metabolites such as IDO and Arg, is associated with the remodeling of immune functions and subpopulations, especially in c-myc or β-catenin-activated HCC.

## 5. Local Metabolic Regulation

In addition to cellular crosstalk within TME, the metabolic landscape is significantly influenced by the anatomical location and overall metabolic condition of patients. The former is determined by liver-specific metabolism and spatial metabolic distinctions within the liver, whereas the latter is determined by various systemic factors, such as diet or gut microbiota [13,61]. Research covering 22 types of tumors revealed that tumor cells exhibit several organ-specific patterns, and that metabolic gene expression is more similar to that in organic tissue [62]. The liver receives blood from both the hepatic artery and portal vein with abundant oxygen and other ingredients. Through the portal vein, the liver is exposed to multiple metabolites from the gastrointestinal tract, enabling the transportation of microbes and microbial and xenobiotic compounds into the liver [63]. After biotransformation and synthesis, manufactured products are transported to the hepatic vein and intestinal tract through systemic circulation and the biliary system [64]. Differential perfusion and cellular composition underlie distinct metabolic conditions, influencing the TME’s immunologic state.

Hepatocytes are commonly separated into two populations based on their distinct metabolic characteristics: periportal and perivenous hepatocytes. Metabolic enzymes involved in carbohydrate or xenobiotic metabolism, ammonia removal, bile transformation, and drug detoxification are preferentially expressed in each zone [61,65]. In the zonation of perivenous hepatocytes, enzymes related to glycolysis, glutamine synthesis, and detoxification are notably upregulated. In addition, enzymes involved in gluconeogenesis and the urea cycle are upregulated in the periportal zone [61,64,65]. This distribution of metabolic enzymes is possibly related to the localization of perivenous β-catenin and the negative regulator Apc in the periportal zone [64,65]. Active signaling by unphosphorylated β-catenin, which promotes glutamine synthesis, is mainly expressed by proximal perivenous hepatocytes and accompanied by the upregulation of β-catenin target genes, including GS, ornithine aminotransferase (Oat), and Glt1. Urea formation, which preferentially occurs in periportal hepatocytes, is decreased in HCC and replaced by glutamine synthesis upon ammonia fixation. This change is accompanied by diffuse GS expression, which is present in perivenous hepatocytes in healthy liver tissues [66]. However, the detailed effects of HCC metabolic zonation on the immune landscape and clinical prognosis must be explored through further research.

## 6. Targeting Metabolism to Restore Immunity

Immunotherapy, including ICIs, virotherapy, vaccination, and adoptive cell therapy (ACT), is effective at enhancing the immune response. Furthermore, the FDA approved the combination of atezolizumab and bevacizumab as a first-line therapy for advanced HCC [4,5]. However, the immunotherapy response rate is still unsatisfactory, partly due to intratumoral heterogeneity and differences in metabolic pathways. In recent years, numerous trials have been conducted to enhance therapeutic efficacy by combining immune checkpoint inhibitors with metabolic interventions [4].

By mediating metabolic processes involving primary nutrients and systemic intervention, enzymes and metabolites are modulated to remodel the metabolic and immunologic landscape of HCC TMEs. Possible connections between distinct metabolic patterns and clinical features have been analyzed through comparisons among specific subtypes [67]. In one study, HCC was divided into three subtypes (iHCC1-3) whose metabolic features and survival rates significantly differed. iHCC1, with its high survival rate and high fluctuations in amino acid, cofactor, and coenzyme metabolism, as well as FAO and OXPHOS, is related to potent inflammatory and immune responses [67]. Increased glutamine metabolism and a low FAS level are associated with CTNNB1 mutation and β-catenin activation in iHCC2 cells [67,68]. Additionally, iHCC3, which has the lowest survival rate, exhibits PI3K/AKT/mTOR activation and high FAS and glycolysis rates. For corresponding clinical strategies to treat HCC, differential metabolic features and diverse signaling pathways must be identified [68].

**Figure 4:**
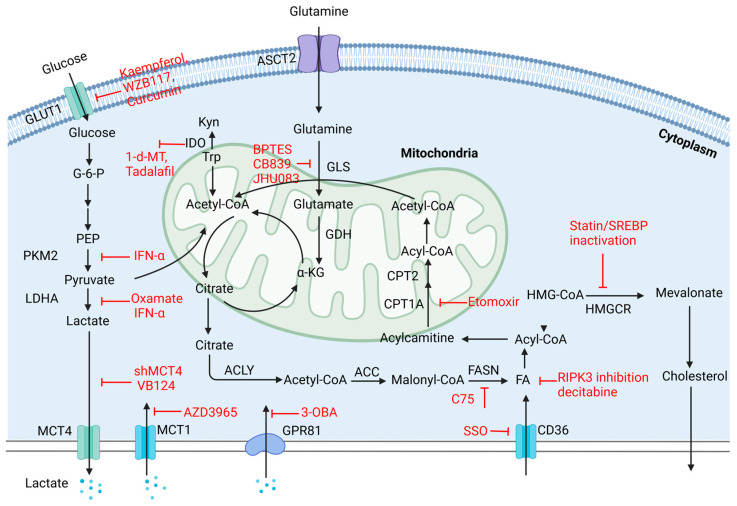
Targeting metabolism to restore immunity. The unsatisfactory response rate to immunotherapy calls for corresponding strategies and accurate targeting of HCC treatment. By regulating the metabolic processes of dominant nutrients or enzymes, the immune landscapes of HCC TMEs are remodeled. 1-d-MT, 1-methyl-d-tryptophan; ACLY, ATP citrate lyase; ASCT2, alanine-serine-cysteine transporter 2; CPT1A, carnitine palmitoyl-transferase 1A; CPT2, carnitine palmitoyl-transferase 2; FA, fatty acid; FASN, fatty acid synthase; G-6-P, glucose 6-phosphate; GDH, glutamate dehydrogenase; GLS, glutaminase; GLUT1, glucose transporter type 1; HCC, hepatocellular carcinoma; HMGCR, 3-Hydroxy-3-methylglutaryl-CoA reductase; IDO, indoleamine 2,3-Dioxygenase; IFN-α, interferon-α; Kyn, kynurenine; LDH, lactate dehydrogenase; PEP, phosphoenolpyruvate; PKM2, pyruvate kinase isozyme type M2; SREBP, sterol regulatory element-binding protein; Trp, Tryptophan.

In this section, we focus on the latest findings regarding possible strategies, mostly in preclinical trials, for modulating immunosuppressive TMEs, improving immune responses, and reversing immune checkpoint resistance by targeting metabolism. The trials mentioned in this article are described in Figure 4.

### 6.1. Targeting Glucose Metabolism

The conversion of glucose metabolism from OXPHOS to aerobic glycolysis is more than a metabolic hallmark of HCC. It also creates promising targets for HCC therapy given the poor response rate to a single agent of immunotherapy [69]. These therapeutic methods mainly include (1) targeting GLUT1 or MCTs to interfere with the transfer of glucose or lactate; (2) targeting critical metabolic enzymes or metabolic intermediates to directly regulate metabolic processes; (3) targeting relevant regulatory molecules or signaling pathways to indirectly influence glucose metabolism. Several examples of each type in recent studies are described below.

Glucose uptake can be downregulated by GLUT1 inhibitors, such as kaempferol, WZB117, and curcumin, which effectively inhibit glycolysis [18]. In addition, MCTs, high-affinity lactate transporters, are highly expressed transmembrane proteins. They assist in the transfer of lactate, pyruvate, or ketone bodies and sustain intracellular pH homeostasis [14]. Among MCTs, MCT1 regulates the entry of lactate and pyruvate, and MCT4 regulates the export of lactate into the TME [70]. MCT4 expression was upregulated in tumor tissues from nonresponders to toripalimab, a PD-1 inhibitor, and CD8+ T-cell recruitment was decreased in a murine model [14]. However, when MCT4 was inhibited via shMCT4 or VB124, a highly potent MCT4 inhibitor, tumor growth was clearly impeded, with increasing infiltration and cytotoxicity of CTLs and glucose flux into the TCA cycle. Owing to their ability to relieve acidification of the TME and increase CXCL9/10 secretion from the NF-κB signaling pathway, MCT4 inhibitors combined with PD-1 inhibition are effective at prolonging mouse survival and have been tested in clinical trials (NCT03867370) [14]. With glucose deprivation of TME due to consumption by tumor cells, Treg cells actively uptake lactate via MCT1, leading to increasing levels of Ca 2+ and NFAT1 translocated into the nucleus. NFAT1, in cooperation with FOXP3, upregulates the expression of multiple immunological molecules, including PD-1 and CTLA-4. Thus, increasing lactate import induces elevated PD-1 expression in Treg cells [20]. The failure of a single PD-1 blockade might result from the immunosuppressive effects of Tregs, which inhibit TCR and CD28. Inhibitors of MCT1 or LDHA effectively reversed ICI resistance in murine models. Moreover, AZD3965, an MCT1 inhibitor, was investigated in a clinical trial (NCT1791595) [20].

Knocking down LDHA decreases the Warburg-like metabolic signature and HCC metastasis in mice [71,72]. The LDHA inhibitor oxamic acid was shown to impede lactate accumulation and synergistically increase the anti-tumor abilities of other drugs, such as sorafenib, in cell line experiments [73]. Moreover, gluconeogenesis suppression is a possible target. Biocompatible nanoparticles have been used as EV mimics to replenish FBP1 (gluconeogenesis-related enzyme) expression in recipient hepatocytes and stimulate PKLR upregulation in hepatocyte-derived EVs, alleviating gluconeogenic suppression, counteracting tumorigenic effects, and enhancing NK cytotoxicity in a diethylnitrosamine-induced mouse model of HCC [34].

In addition, in terms of immunometabolites, itaconate has been widely researched for its clinical effects. In one study on a mouse model of HCC, low-dose (20 mg/kg) ibuprofen attenuated the epigenetic connection between macrophage metabolism and CTL exhaustion by reducing IRG1 (itaconate-synthesizing enzyme) expression and itaconate synthesis, alongside an apparent anti-tumor effect [28]. In a mouse model of breast cancer, IRG1 and itaconate were upregulated in immunosuppressive tumor-infiltrating neutrophils (TINs) to survive in metastasis by activating the Nrf2-mediated antioxidant response to escape ferroptosis [29]. The specific neutrophil ablation of IRG1 in Mrp8-cre+IRG1f/f mice decreased TIN survival and delayed metastasis growth, prolonging animal survival. In addition, the loss of IRG1 recovered T-cell anti-tumor functions and enhanced the therapeutic effects of ICB [29]. Similarly, in models of hematologic malignancies, STAT3-signaling-dependent itaconate production, activates Nrf2, antioxidant mechanisms, and MDSC survival, decreasing anti-tumor immune responses. By blocking β2-AR, STAT-3 signaling, or itaconate metabolites, impairing the immunosuppressive functions of MDSCs is possible [74]. In conclusion, these findings suggest that itaconate and IRG1 might be valuable immune–metabolic targets across tumors, including HCC, to elevate immune responses.

The highly hypoxic and acidic conditions of the TME propel the activation of the HIF-1α signaling pathway, which could remodel the immune landscape of the TME. HIF-1α upregulates glycolysis and the PI3K/AKT/mTOR pathway, promoting ICI resistance [75]. However, by inducing IRF1 expression and inactivating downstream FosB transcription, IFN-α attenuates HIF-1α transcription and decreases glycolysis-related gene expression [11]. By combining IFN-α and PD-L1 blockades, remodeled glucose metabolism fosters a comparatively high-glucose TME with upregulated CD27 transcription, cytotoxicity, and an effective immune response. According to data from a murine model and clinical data from 15 patients with HCC, the combined administration of IFN-α and PD-L1 blockades led to notable tumor regression, providing promising evidence for the translational and clinical application of this combination treatment [11]. Moreover, lactate triggers tumoral PD-L1 expression by activating the lactate receptor and GPR81 and HIF-1α signaling pathways, reducing intracellular cAMP and PKA activity. The dual blockade of GPR81 by its antagonist, 3-hydroxy-butyrate (3-OBA) and the PD-1/PD-L1 pathway profoundly enhances CTL infiltration and IFN-γ secretion, resulting in tumor regression in animal models in vivo [76].

In general, alterations in glucose metabolism significantly influence HCC progression, including proliferation, immune escape, or drug resistance. By targeting glucose or lactate transporters, critical metabolic enzymes or intermediates, and regulatory factors or signaling pathways in combination with immunotherapy, various novel therapeutic methods have proven effective in experiments involving HCC cell lines or animal models. Although some of these drugs have been incorporated into phase I or II clinical trials, their safety and effectiveness for clinical application are still unclear, calling for further evaluation of tumor and personal heterogeneity.

### 6.2. Targeting Lipid Metabolism

The roles that lipid metabolism plays in liver carcinogenesis are versatile and complex across different subtypes, the alterations of which are highly heterogeneous and closely associated with clinical characteristics. In a study analyzing serum lipids, plasmalogens, a special type of phospholipid, differentiated HCC patients according to clinical grade [77]. Moreover, HCC steatosis might be a novel imaging biomarker. Steatotic HCC is characterized by an immune-enriched but exhausted TME. HCC patients with MRI-identified steatotic livers may be more susceptible to immunotherapy with high CTL infiltration and PD-L1 expression [38,78].

In addition, as a vital energy source, lipid uptake, synthesis, and oxidation, as well as cholesterol metabolism, are closely related to the metabolic and immune conditions of the cellular composites of HCC TMEs. For instance, the coculture of CD36+CAFs with HCC cells accelerates tumor progression, while combined treatment with sulfosuccinimidyl oleate (SSO, an irreversible inhibitor of FA translocase CD36 that significantly restrains FA import) and ICI is known to be effective at restoring the immunity of T-cells, partly owing to the poor metabolic flexibility of tumor cells compared to T-cells [45]. In addition to sustaining lipogenesis, PA can mediate oncogenic AKT activation through palmitoyl modification catalyzed by the palmitoyl-transferase ZDHHC17/24. Therefore, by restricting PA intake from the diet or utilizing a FASN inhibitor, orlistat, to decrease PA synthesis after the TCA cycle, palmitoyl modification and activation of AKT are significantly attenuated, resulting in weakened oncogenesis [79]. Similar results were observed when small peptides were used to compete with ZDHHC24-regulated AKT palmitoylation and activation, suggesting that PA intake, synthesis, and ZDHHC17/24 might be novel epigenetic biomarkers and therapeutic targets for HCC patients [79]. In addition, FASN ablation by its inhibitor C75 delays but does not completely inhibit tumorigenesis in mice, possibly owing to the compensatory upregulation of HMGCR expression (3-hydroxy-3-methylglutaryl-CoA reductase, a critical metabolic enzyme to cholesterol synthesis) and cholesterol biosynthesis, which can be remedied by the dual blockade of FASN and HMGCR. Through experiments using siRNAs against FASN and HMGCR in a murine model of HCC, cellular growth was inhibited, consistent with previous findings on statins’ potential anti-tumor role [80].

In terms of FAO, IL4-mediated M2 polarization is counteracted when FAO is inhibited by etomoxir (a clinically approved FAO inhibitor) or CPT1A shRNA [81]. When decitabine is used to sustain RIPK3 (a vital factor in inflammation and necroptosis) hypomethylation or FAO targeting in RIPK3-KO mice, the anti-tumor immunity of TAMs is elevated and M2 polarization is attenuated, resulting in FA metabolism suppression [44]. In addition to FAs, other types of lipids, such as phospholipids, also function in dysregulated lipid metabolism. Phospholipase A2 Group VII (PLA2G7), a member of the phospholipase A2 family, mediates phospholipid hydrolysis and discharges FAs and lysophospholipids [82]. PLA2G7-high macrophages exhibit immunosuppressive features and attenuate T-cell activation. By pharmacologically inhibiting PLA2G7 in vivo or by co-culture in vitro, PLA2G7 inhibition via darapladib promoted NF-κB pathway-mediated M1 TAM infiltration and sensitized HCC cells to ICB therapy [83].

In a novel study, nanodrugs were injected intratumorally to capture tumor-associated antigens (TAAs) and address the poor immune response mediated by insufficient antigen internalization from combined treatment with ICB and thermal ablation [84]. Upon the capture of released TAAs, nanodrugs were endocytosed into DCs containing TAAs and m6A demethylases inhibitor (also referred to as fat mass and obesity-associated gene (FTO) inhibitor). In an HCC-bearing mouse model, the nanodrug succeeded in driving DC maturation, antigen presentation, and anti-tumor immunity [84].

Therefore, due to the complexity and flexibility of lipid metabolism, lipid-targeting drugs and strategies are mostly used in preclinical studies. Comprehensive consideration of these drugs’ interaction with the TME is necessary for assessing their reliability for clinical application.

### 6.3. Targeting Amino Acid Metabolism

As another source of energy, amino acid metabolism, especially glutamine metabolism, is used to synthesize biological macromolecules, during which its intermediates are also raw ingredients for synthesizing FAs, proteins, and nucleotides. As the most abundant type of amino acid in the muscle and plasma of humans, the vital role of glutamine metabolism during carcinogenesis has been demonstrated by both in vivo and in vitro experiments, which have prompted diverse studies exploring potential drugs for clinical application [85,86].

Recent studies have focused on the initial step of glutamine metabolism, GLS-mediated glutamine catabolism. Due to the distinctive functions of GLS1 and GLS2, with GLS1 related to upregulated carcinogenesis and GLS2 linked to differentiated or quiescent cellular states, different strategies have been proposed [63]. By restoring GLS2 to arrest the cell cycle in the G2/M phase and inhibiting GLS1 to impede tumorigenesis, small molecule inhibitors to attenuate GLS1, including Bis-2-(5-phenylacet-amido-1,3,4-thiadiazol-2-yl) ethyl sulfide (BPTES) and CB839 (BPTES derivatives), have been developed [87]. However, due to their poor water solubility and low binding selectivity and bioavailability, most of these drugs have not been incorporated into clinical research. To solve these problems, prodrugs, which are delivered to tumor tissues by nanocapsules such as JHU083 (a DON prodrug used for tumor-targeted glutamine inhibition) have been developed to selectively inhibit glutamine metabolism [85]. In a subcutaneous tumor-forming mouse model, when JHU083 was used to inhibit glutamine metabolism, CD8+T-cell proliferation was promoted with dampened ICI resistance, partly due to enriched exogenous lipid metabolism and lipolysis-originated ketone bodies that alternatively replenished energy stores [55,56].

Glutamine addiction commonly occurs in tumors, especially when it is driven by c-myc mutations and mTOR activation, which increase HCC cells’ metabolic dependence on exogenous glutamine and elevated glutamine catabolism. C-myc-induced glutamate-oxaloacetate transaminase 1 (GOT1) expression and Keap1-Nrf2 signaling pathway activation upon glutamine starvation facilitate GSH synthesis and rescue tumor cells from ROS and ferroptosis, indicating that additional GOT1 knockout combined with glutamine restriction can cause fatal damage to tumor cells [88]. Notably, there were therapeutic benefits (decreased tumor metabolism and cellular proliferation) after inhibiting mTORC1 in β-catenin-mutated HCC [54]. Inhibiting mTOR alone or in combination with Met rescued effector T-cell function and reduced the HCC burden. Supplementation with Met also promoted IL-2, IFN-γ, and TNF-α secretion from CTLs and restored immunity in preclinical models [89]. In addition, epigenetic RNA-RNA crosstalk between HMGB1 and RICTOR has been shown to regulate glutamine metabolism, which activates TLR4 and GS via the mTORC2-AKT-c-myc pathway. When this crosstalk is interrupted, a potent immune response toward ICIs was shown to occur in a murine model of HCC due to the inhibition of mTORC1-dependent PD-L1 production and the presence of PD-L1+ exosomes, suggesting that metabolic intervention by epigenetic regulation, such as targeting HMGB1, can also be a novel therapeutic target for ICI resistance [49,90].

In addition to glutamine, kynurenine also propels immunosuppressive TMEs, mainly by upregulating IDO, degrading tryptophan, and promoting adaptive resistance. ICI resistance originates from an IFN-γ-mediated increase in IDO in anti-CTLA-4-treated HCC cells and can be reversed by the IDO inhibitor 1-methyl-d-tryptophan (1-d-MT), as demonstrated in animal experiments [91]. Since HCC tumor cells highly rely on extracellular Arg instead of internal production for survival, novel strategies for triggering cellular autophagy or apoptosis via external Arg depletion have been developed [92]. In recent studies, Arg depletion has mostly been achieved by the development of Arg-degrading enzymes, including Arg1, Arg decarboxylase, and deiminase. The positive results of several preclinical and early-stage clinical studies using ADI-PEG 20 and rhArg1 to degrade Arg suggest the promising effectiveness of Arg depletion therapy [58,92]. However, the broad limitations of circulating Arg negatively affect T-cell activation, which can be avoided by targeting the cancer-specific arginine-binding factor RBM39 [57]. Experiments on cells, mouse models, and patient-derived organoids indicate that molecular glues, like aryl sulfonamide indisulam, which specifically target the ubiquitination and degradation of RBM39, could benefit HCC patients with decreasing levels of tumoral Arg and RBM39 expression [93]. Tadalafil (TA), which is used to treat erectile dysfunction and pulmonary hypertension, has been effective at offsetting ICI resistance by repolarizing macrophages to the M1 phenotype and causing Arg1 deprivation in MDSCs [94]. Based on these findings, a tumor-targeted nanocarrier encapsulating anti-PD-1 and TA was created and delivered to an orthotopic HCC model. This nanocarrier blocks the PD-1/PD-L1 axis to restore CTL cytotoxicity, after which TA is transported into TAMs and MDACs upon release of the lysosomal drug in vitro and in orthotopic HCC models, respectively [95]. This innovative form of drug targeting could augment ICI efficacy and minimize side effects.

In conclusion, restricting tumor cells’ amino acid availability and sustaining the supply of normal tissues could improve therapeutic outcomes. However, despite the development of multiple strategies to target amino acid metabolism, amino acid dependence levels are different, which necessitates the identification of biomarkers to differentiate specific levels of metabolic reliance and determine whether patients would benefit from treatments targeting amino acid metabolism.

### 6.4. Targeting Systemic Metabolism

Due to the anatomical and functional links between the liver and gut, interactions between diet, gut microbiota, and immunity significantly affect HCC progression [96]. Gut microbiota profoundly influences HCC TME when metabolites enter the systemic circulation and bind to specific receptors [63]. Obesity or extra-calorie consumption is related to a greater risk of carcinogenesis, whereas a low-carbohydrate and low-fat diet is associated with a lower tumor incidence [97]. In experiments on a mouse model of HCC, two diets, “low-carb high fat” and “low-carb high protein”, were found to retard tumor progression and prolong survival [96]. Although dietary intervention can potentially affect prognosis and survival, it is unlikely to result in tumor regression by itself, possibly because nutrient restriction mainly modulates cellular proliferation instead of survival. Dietary intervention is assumed to function by mediating metabolic levels in plasma and the TME, altering the accessibility and composition of nutrients, and influencing the functional conditions of cellular components and therapeutic responses [97]. Here, we introduce several typical dietary intervention patterns.

Ketogenic diets (KDs), which are low in carbohydrates and high in fat, cause broad changes in systemic nutrient availability and utilize ketone bodies as alternative energy sources. A KD is a potential cancer therapy because it decreases circulating glucose levels and activates growth-stimulating insulin signaling [96,98]. However, ketogenic diet response might be genotype-dependent. For instance, KD-induced ketogenesis results in the accumulation of ketone bodies, enhanced BRAF signaling, and BRAF V600E melanoma progression [98]. A realist review revealed that 10 out of 24 (42%) clinical studies supported the positive role of KDs in preventing tumors, seven of which lacked significant differences and one reported negative effects [98,99].

Moreover, high-fat high-cholesterol (HFHC)-fed mice sequentially developed NAFLD-HCC accompanied by insulin resistance, and high-fat low-cholesterol diet-fed mice simply developed hepatic steatosis. Cholesterol-mediated HCC generation occurs simultaneously with dysbiosis of the intestinal microbiota, along with hepatic ROS and intrahepatic infiltration of NKT cells and inflammatory cytokines [100]. Stool from HFHC-fed mice revealed that lipids accumulated, cells proliferated, and inflammation increased in the livers of germ-free mice. However, anti-cholesterol therapies, such as atorvastatin, can ameliorate microbiota dysbiosis and inhibit the progression of NAFLD-related HCC, indicating cholesterol’s specific role in liver carcinogenesis [100].

In addition, the gut microbial community, which colonizes the body and produces a series of metabolites, can be regulated by dietary intake and indirectly influence cellular metabolism and the immune response [13,63]. In preclinical mouse models and observational cohorts of patients, intestinal microbiota has been shown to mediate immunotherapy response [101]. In an analysis of fecal samples from patients receiving ICIs, taxa richness and gene counts were greater in responders [102]. Microbial dissimilarity was also detected in the fecal microbiota between responders and nonresponders among patients with unresectable HCC [7]. According to human research on melanoma, patients who were responsive to ICIs had a greater diversity of fecal microbiota and a greater abundance of Ruminococcaceae. Accordingly, feeding mice stool from responders enhanced tumor control, upregulated T-cell responses, and improved immunotherapy efficacy [103]. Since the interplay between microbiota and immune checkpoints can lead to immune escape, targeting gut microbiota via methods such as fecal microbiota transplantation (FMT), probiotics, or antibiotics is under investigation [104]. Several clinical trials have been performed to evaluate the efficiency, safety, and feasibility of combining FMT and PD-1 inhibitors. In one trial, three out of ten (30%) metastatic melanoma patients achieved clinical responses and tumor regression. One achieved a complete response and two achieved partial responses after receiving FMT from responders and reinitiating anti-PD-1 therapy [7,101]. In addition, probiotics can maintain the gut microbial balance, sustain the integrity of the intestinal barrier, and prevent leakage and migration of microbial metabolites into circulation.

Although diet-mediated changes in systemic metabolism and nutrient availability can alter metabolic state, detailed pathways through which dietary interventions mediate immune responses are mostly unclear. Research on nutrient distribution and perfusion, specific metabolic heterogeneity or vulnerabilities, and further clinical trials are crucial for guiding clinical decision-making [13,97].

## 7. Conclusions

During the initiation and progression of HCC, dynamic changes in the metabolism of the TME not only influence the cellular availability of nutrients and energy stores through crosstalk and competition between cellular components but also modulate the immune landscape and response to immunotherapy. With rapid advances in metabolite qualification and quantification technology, gaining a comprehensive understanding of the metabolome in biological samples and analyzing alterations in metabolites and metabolic pathways is possible. On this basis, versatile drugs have been developed to target the dysregulated metabolism of HCC TMEs. However, due to the variability and heterogeneity of HCC patients’ metabolic conditions, research into several aspects is still needed, including biomarker identification to differentiate potential HCC patients for metabolic intervention therapy, drug interactions with HCC TMEs, the regulation of compensatory pathways, as well as accurate targeting of specific cellular metabolism pathways to avoid side effects on other cells. Through the recognition and targeting of distinct metabolic vulnerabilities in HCC cells, HCC patients could benefit from combined metabolic intervention and immunotherapy with improved clinical outcomes.
